# The British Society for Rheumatology Biologics Registers in Ankylosing Spondylitis (BSRBR-AS) study: Protocol for a prospective cohort study of the long-term safety and quality of life outcomes of biologic treatment

**DOI:** 10.1186/s12891-015-0805-x

**Published:** 2015-11-11

**Authors:** Gary J. Macfarlane, Maxwell S. Barnish, Elizabeth A. Jones, Lesley Kay, Andrew Keat, Karen T. Meldrum, Ejaz Pathan, Roger D. Sturrock, Claudia Zabke, Paul McNamee, Gareth T. Jones

**Affiliations:** Musculoskeletal Research Collaboration (Epidemiology Group), Institute of Applied Health Sciences, University of Aberdeen, Aberdeen, UK; Department of Rheumatology, Freeman Hospital, Newcastle upon Tyne, UK; Rheumatology Department, Northwick Park Hospital, London, UK; Department of Rheumatology, Aberdeen Royal Infirmary, Aberdeen, UK; Division of Immunology, Infection and Inflammation, University of Glasgow, Glasgow, UK; Centre for Rheumatic Diseases, Glasgow Royal Infirmary, Glasgow, UK; Health Economics Research Unit, Institute of Applied Health Sciences, University of Aberdeen, Aberdeen, UK

**Keywords:** Axial spondyloarthropathy, Ankylosing spondylitis, Biologic therapy, Safety, Infection, Cohort

## Abstract

**Background:**

Axial spondyloarthropathy typically has its onset in early adulthood and can impact significantly on quality of life. In the UK, biologic anti-tumour necrosis factor therapy is recommended for patients who are unresponsive to non-steroidal anti-inflammatory drugs. There remain several unresolved issues about the long-term safety and quality of life outcomes of biologic treatment in axial spondyloarthropathy. Long-term “real-world” surveillance data are required to complement data from randomised controlled trials.

**Methods/Design:**

We are conducting a UK-wide prospective cohort study of patients with axial spondyloarthropathy who are naïve to biologic therapy at the time of recruitment. Those about to commence anti-tumour necrosis factor biologic therapy will enter a “biologic” sub-cohort with other patients assigned to a “non-biologic” sub-cohort. The primary objective is to determine whether the use of biologic therapy is associated with an increased risk of serious infection, while secondary objectives are to assess differences in malignancy, serious comorbidity, all-cause mortality but also assess impact on specific clinical domains (physical health, mental health and quality of life) including work outcomes between biologic and non-biologic patient cohorts. Patients will be followed-up for up to 5 years. Data are obtained at baseline and at standard clinical follow-up visits – at 3, 6 and 12 months and then annually for the biologic cohort and annually for the non-biologic cohort. This study will also collect biological samples for genetic analysis.

**Discussion:**

Although biologic therapy is widely used for ankylosing spondylitis patients who are unresponsive to non-steroidal anti-inflammatory drugs, the majority of the available safety information comes from rheumatoid arthritis, where increased infection risk has consistently been shown. However, given the typical demographic differences between rheumatoid arthritis and axial spondyloarthropathy patients, it is important to develop an epidemiologically rigorous cohort of patients receiving biologic therapy to effectively evaluate outcomes with regard not only to safety but also to quantify benefits across clinical, psychosocial and work outcomes.

**Clinical trial registration:**

This is an observational cohort study and clinical trial registration was not required or obtained

## Background

Axial spondyloarthritis (axSpA) is a chronic inflammatory condition that principally affects the spine and sacroiliac joints but is also associated with peripheral arthritis plus various extra-articular features such as enthesitis, iritis, inflammatory bowel disease and psoriasis. There are two sub-groups *–* radiographic axSpA, also known as ankylosing spondylitis (AS), and non-radiographic axSpA – which have been shown to have similar clinical manifestations and disease activity measures [1]. AxSpA typically has its onset early in adulthood [2] and can have a significant impact on aspects of quality of life including disability and work status [3]. Until recently, treatment has largely been limited to non-steroidal anti-inflammatory drugs (NSAIDs) and physiotherapy, while disease-modifying antirheumatic drugs (DMARDs), though effective in other inflammatory arthritides, have shown little efficacy in axSpA. The introduction of anti-tumour necrosis factor (anti-TNF) biologic therapies has been associated with significantly improved outcomes, including improvements in pain, stiffness and fatigue [4, 5]. Anti-TNF therapy was initially recommended in the United Kingdom (UK) for patients with radiographic axSpA who were unresponsive to NSAIDs and who met specific disease severity criteria [6] and the extension of this indication to non-radiographic axSpA is being considered [7].

Research has highlighted the importance of genetics in axSpA, in particular the HLA-B27 allele [2]. However, many important genetic pathways remain unknown and their relationships to the development of axSpA unconfirmed. Genome-wide genetic association studies are currently underway in international collaborations to elucidate these pathways and the roles they play in axSpA pathogenesis. Although data from randomised clinical trials (RCTs) of biologic therapy in axSpA have been highly informative, there remain a number of unresolved issues, since RCTs are conducted typically on more highly selected populations than are representative of routine clinical practice [8].

Pharmacological management in rheumatology, including the use of disease-suppressive agents, is associated with adverse side effects in a proportion of patients. In addition, longer term complications, such as malignancy, are unlikely to be detected in the relatively short follow-up period of RCTs. Immunosuppressive therapy, in particular, is considered to be a potential risk factor for both malignancy and life-threatening infection. The use of azathioprine and cyclophosphamide, for example, is associated with an increased risk of lymphoproliferative malignancies in patients with rheumatoid diseases [9–11]. Immunosuppressed patients are also at risk of serious infections such as from *Mycobacterium tuberculosis*, *Pneumocystis carinii* and fungal infections [12].

Informed prescribing of biologic agents requires knowledge of the magnitude of risk of such longer-term adverse events. To date, most of the safety information regarding anti-TNF therapy has come from rheumatoid arthritis (RA). It is problematic to extrapolate from one condition to another: RA patients generally have older age at onset than axSpA patients, have a significant burden of co-morbidity, have a longer history of medication use and have often received polypharmacy including many other immunosuppressive drugs such as corticosteroids [2, 13–15]. In terms of benefit, the impact of biologic therapy on employment maintenance and long-term quality of life is of great significance for patients and real-world data are needed in this respect. Furthermore, the ability to identify those who are most likely to benefit from a particular treatment is crucial in delivering cost-effective care. In order to provide axSpA-specific data on the long term safety and benefit of biologic therapy, we are conducting a nationwide prospective cohort study: the British Society for Rheumatology Biologics Register in Ankylosing Spondylitis (BSRBR-AS).

## Methods/Design

### Cohort population

Participants are required to meet the Assessment of SpondyloArthritis international Society (ASAS) criteria for radiographic or non-radiographic axSpA, i.e they must satisfy at least one of the following: i) the modified New York criteria for AS [16]; ii) the imaging-based ASAS definition of axSpA [17]; or iii) the “clinical” ASAS definition of axSpA [17]. At the time of recruitment they are required to be naïve to biologic therapy. Individuals starting an “eligible” biologic therapy comprise the “biologic” cohort and those not starting a biologic therapy join the “non-biologic” cohort. The eligible drug list currently comprises adalimumab (Humira), etanercept (Enbrel) and certolizumab pegol (Cimzia). Participants must be aged at least 16 years and be willing to give informed consent for follow-up including access to medical records. Study exclusion criteria are: i) otherwise eligible patients who are starting a biologic therapy not on the eligible drug list and ii) inability to communicate in English. However, once recruited to the study, participants are eligible to remain in the study irrespective of the subsequent pharmacological or non-pharmacological management of their condition. Recruitment can take place through participating National Health Service (NHS) hospitals across the UK.

### Study procedures

Patients are informed about BSRBR-AS prior to their clinic appointment by means of a letter from their consultant rheumatologist and provided with an information sheet. When patients attend their routine clinic appointment, they are asked whether they would like to take part in the study. Consent is taken by the consultant rheumatologist or an appropriately trained member of the site research team. In exceptional circumstances, patients may complete the consent form at home and return it by post. Patients are ordinarily given at least 24 hours to consider the Patient Information Sheet and are given the opportunity to ask questions before deciding whether to take part.

All participants are assessed at time of recruitment (baseline). Patients in the biologic cohort are followed up at 3, 6 and 12 months after their first dose of biologic therapy and then annually thereafter. Patients in the non-biologic cohort are followed up annually from study entry. This is in line with current UK clinical practice. Each follow-up involves the collection of clinical and self-report data as detailed in the section ‘data collection and management’. Recruitment began in December 2012 and is currently scheduled to continue until December 2016 and follow-up will continue to the end of the study period (currently December 2017), which will provide up to 5 years follow-up for each participant. Assessment of endpoints will be achieved using a combination of physician and patient report and record linkage to routinely collected health data.

### Data collection and management

Clinical data are collected by site clinicians, research nurses or appropriate delegate at routine clinical appointments. All data are entered onto the electronic Case Report Form (eCRF).

The following clinical measures are recorded:

i) Eligibility
ii) Targeted medical history, blood pressure, weight and height
iii) Active phase reactants (serum C reactive protein (CRP) or if not available erythrocyte sedimentation rate (ESR))
iv) Bath Ankylosing Spondylitis Metrology Index (BASMI) [18]
v) Extra-articular disease features
vi) Pregnancy status of patient or partner

The following self-report measures are collected:

i) Demographic information
ii) Current smoking status and alcohol consumption
iii) Disease activity:
  ○ Bath Ankylosing Spondylitis Disease Activity Index (BASDAI) [19]
  ○ Ankylosing Spondylitis Disease Activity Score (ASDAS) [20]
iv) General measures of AS:
  ○ Bath Ankylosing Spondylitis Global Score (BAS-G) [21]
  ○ Bath Ankylosing Spondylitis Functional Index (BASFI) [22]
  ○ Spinal pain visual analogue scale
v) Quality of life:
  ○ Short Form Health Survey (SF-12) [23]
  ○ Ankylosing Spondylitis Quality of Life Index (ASQoL) [24]
  ○ EuroQoL quality of life measures (EQ-5D-5L and EQ-VAS) [25]
vi) Other measures including:
  ○ Chalder fatigue scale [26]
  ○ Estimation of Sleep Problems [27]
  ○ Hospital Anxiety and Depression Scale (HADS) [28]
  ○ Work Productivity and Activity Impairment Questionnaire: Specific Health Problem (WPAI: SHP) [29]
  ○ American College of Rheumatology (ACR) 2011 research criteria for fibromyalgia [30]

### Health economics

The health economics analysis will involve the construction of cost and quality of life profiles, based on EQ-5D-5L and SF-6D (derived from SF-12), for different axSpA health states. Costs will include the number of visits to NHS hospitals for inpatient treatment, day-case procedures and outpatient services associated with the condition and related side-effects, recorded from routine records, valued using standard national sources such as NHS Reference Costs [31]. Quality of life profiles will be estimated from patient self-report, and valued using published UK tariffs. The estimated cost and quality of life profiles will facilitate the development of economic models to assess the cost-effectiveness of biologic therapy versus non-biologic therapy. A unique feature of these profiles is that they will be developed from real-life registry data, and so are more likely to be generalisable to NHS patients than previously published estimates.

### Pharmacovigilance and safety reporting

Clinically confirmed Serious Adverse Events related to biologic therapies on the eligible drug list are reported to the relevant pharmaceutical company.

### Data analysis and statistics

The study was powered on the primary objective – an ability to determine a doubling of the risk of serious infection, i.e infection resulting in hospitalisation or death, among axSpA patients treated with biologics compared to the non-biologic cohort. Analysis of this primary endpoint will be based on comparing the risks of events over time using Cox proportional hazards regression [32], taking into account differences between groups such as potential confounders and disease modifying effects. We will control for medication switching in the analysis.

Available data from a meta-analysis of persons enrolled in trials suggest that the baseline risk of serious infection is 1.0 per 100 person years in those not randomised to biologic therapy and that there is an approximate doubling in risk in those receiving biologics [33]. Noting that adverse events will be more common in routine practice we have powered the study on a baseline risk of 1.6 cases per 100 person-years. The power calculation is taken as a time-to-event analysis and is based on an expected ratio of 35 biologic patients to every 65 non-biologic patients. Assuming we wish to be able to detect a Hazard Ratio (HR) of 2 in the biologic cohort compared to the non-biologic cohort, i.e. an event rate of 3.2 per 100 person years, the number of events required for 80 % power is 7.9/(0.65 * 0.35 * log (HR) ^ 2) = 73 events [34], which will be provided by 1184 and 2216 person-years of observation in the biologic and non-biologic cohorts respectively. Considering alternative outcomes, not necessarily related to safety, but also for example treatment response, improvements in symptoms and quality of life, power increases to >95 % for baseline hazards of 3 per 100 person-years in detecting a HR of 2. For baseline hazards of 10 per 100 person-years the study has 95 % power to detect a HR of 1.5 while the study has 80 % power to detect a HR of 1.2 for baseline hazards of 30 per 100 person-years.

### Study management and oversight arrangements

The study is co-ordinated by a Study Management Group consisting of the study investigators. A study co-ordinator oversees the study and is accountable to the Chief Investigator. An International Advisory Committee advises on maximising the usefulness of the register for research purposes and on potential collaborations, harmonising opportunities for data collection with other international studies. A Data Monitoring Committee is convened by the British Society for Rheumatology (BSR). The register and data belong to the BSR and oversight of this register (and other registers of the BSR) is undertaken by the BSR Registers Committee. The committee membership comprises BSR clinical affairs staff, representatives from BSR members and from the study investigators.

### Research governance

This is an observational cohort study and clinical trial registration was not required or obtained. The study was peer-reviewed as part of the process of applying for funds, competitively, to the BSR. Ethical approval was obtained from the National Research Ethics Service (NRES) Committee North East – County Durham and Tees Valley (Research Ethics Committee (REC) reference 11/NE/0374). Appropriate NHS Research and Development (R&D) approvals are obtained for each site prior to the commencement of study operations in that location. The BSR is the legal sponsor for the study and delegates certain functions to the University of Aberdeen. All clinical sites will only have access to local identifiable patient information. All patient identifiable study information will be kept confidential and in compliance with the Data Protection Act (1998).

### Dissemination of findings

Dissemination activities in BSRBR-AS will comply with the BSR Publication Policy. Summaries of results will be made available for investigators to disseminate within their clinical areas and reports will be produced for the sponsor, REC and NHS R&D.

## Discussion

This study represents one of only a small number of registers focused on biologic therapy among persons with axSpA. Such therapies have been shown to be effective in clinical trials in achieving ASAS 50 % response, in improving disease activity and function [35]. So why the need for a register? Firstly, trials are conducted on highly selected populations [8]. Specifically, trial populations tend to be younger and have fewer comorbidities than the general patient population. Trials focus on evaluating efficacy, are short-term and conducted on relatively small numbers of patients. In contrast, this register is recruiting patients with axSpA from centres throughout the UK. These include some centres with specific expertise in managing axSpA, but most are local hospitals responsible for managing all rheumatic diseases arising in patients within their local population. Secondly, eligibility for the register is determined, in terms of disease status, only in relation to having an eligible diagnosis and in being naïve to biologic therapy. Thus, patients are not excluded on the basis of multi-morbidity. Thirdly, patients will be followed up until the end of study, providing longer-term follow-up. This will then allow examination of both effectiveness, impact and safety of these therapies over the medium term.

A novel aspect of the register is the inclusion of persons who meet only the clinical arm of the ASAS criteria for axSpA, namely patients who are HLA-B27 positive and have 2 additional SpA features, but who do not meet the imaging arm of the ASAS criteria. This will allow the natural history of axSpA in this sub-group to be determined. Indeed, the lack of information on the natural history of non-radiographic axSpA was highlighted by the US Food and Drug Administration (FDA) in its ruling against widening indications for anti-TNF therapy beyond radiographically confirmed axSpA [36]. The current register is the second under the auspices of the BSR. The first, the British Society for Rheumatology Biologics Register in Rheumatoid Arthritis (BSRBR-RA), was established over 10 years ago. It uses a similar design as BSRBR-AS and has provided important evidence on safety of anti-TNF therapy in that population. For example, the addition of anti-TNF therapy to DMARDs did not alter the risk of cancer in RA patients selected for anti-TNF therapy [37]; a significantly increased risk of shingles and a doubling in risk of septic arthritis was observed in those treated with anti-TNF therapy but an observed increased risk of serious skin and soft tissue infections was not statistically significant [38, 39] and there was no excess risk of venous thromboembolism [40]. Using BSRBR-RA data, Rituximab has been shown to be a more effective option than a second anti-TNF therapy for patients who have not responded to an initial anti-TNF therapy [41].

In 2015, the first ‘biosimilars’ for RA, axSpA and psoriatic arthritis (PsA) were introduced to the market. A biosimilar is a biological medicine manufactured to be similar to an existing ‘reference’ biological medicine, with no meaningful differences in terms of safety or efficacy. Although pre-market authorisation includes comparisons against the reference medicine, this typically involves small scale trials and they are not undertaken for all indications of the reference product. There is also little evidence on the safety and efficacy of switching to biosimilars – and it is thought that many patients may have their therapy switched in order to reduce costs to the NHS. The BSR has therefore recommended strongly that all patients starting or switching to biosimilar agents should be registered with the appropriate biologics register. The current study will seek to capture such information on participating patients within BSRBR-AS, thus expanding the evidence base for these agents [42].
